# Feasibility of Imaging the Uvula at the Midtrimester Anomaly Ultrasound: A Retrospective Study

**DOI:** 10.1002/jum.70221

**Published:** 2026-03-08

**Authors:** Anna Rose Sims, Lou Pistorius, Denise Sproul, Catherine Anne Cluver

**Affiliations:** ^1^ Department Obstetrics and Gynecology University of Stellenbosch Cape Town South Africa; ^2^ Panorama Perinatology Mediclinic Panorama Cape Town South Africa

**Keywords:** equal sign, palate, screening, ultrasound, uvula

## Abstract

**Objectives:**

The fetal palate is not routinely imaged as part of the midtrimester fetal anomaly ultrasound, despite being associated with many syndromes. The “equal sign” depicts the lateral borders of the uvula on 2‐dimensional fetal ultrasound. We assessed the feasibility of adding the equal sign to the midtrimester fetal anomaly ultrasound.

**Methods:**

A retrospective audit of an ultrasound practice in Cape Town, South Africa was performed. Women with singleton pregnancies presenting for a routine anomaly ultrasound between October 2013 and September 2019 were included.

**Results:**

A total of 5301 ultrasound examinations were performed during the time period. In a sample of 101 cases selected randomly, the equal sign was clearly visualized in 97% (98/101) of cases. It was visualized in sequence while examining the face or brain in 88% (89/101). Mean time to visualize the equal sign was 28 seconds. Total scan time increased by 1.5%. The body mass index had no effect on detection rate. Of the 5301 scans performed during the study period, there were 15 cases where the uvula was abnormal. Ten of these 15 cases had an intact lip. There were no false positive diagnoses of an abnormal uvula.

**Conclusions:**

It is feasible to image the equal sign at the midtrimester ultrasound with a low false positive detection rate.

**Abbreviations**
AIUM: American Institute of Ultrasound in Medicine
BMI: body mass index
ISUOG: International Society of Ultrasound in Obstetrics

Oral clefts are a common malformation which include cleft lips, cleft palates and a combination of cleft lip and palate. The prevalence varies with an estimate of 6.4 per 10 000 live and stillbirths^1^ For isolated cleft palate the incidence varies substantially by geographic location and has been reported to be between 1.3 and 25.3 per 10 000 live births.^2^ The highest rates of isolated cleft palate have been reported in Canada and the lowest rates in Africa.^2^ Females have consistently higher rates of isolated cleft palates.^2^ Most oral clefts are non‐syndromic except for cleft palates. Syndromes have been identified in about half of cases with cleft palates and include over 100 different syndromes.^3^

Prenatal detection of oral clefts is becoming more relevant as prenatal genetic testing options like microarrays and whole exome sequencing are more readily available. Prenatal diagnosis provides time for parents to become informed of the implications of an orofacial cleft and allows them to consider prenatal testing. In some cases the option of termination of pregnancy may be considered. Prenatal diagnosis also provides time to prepare for the needs of a child with an orofacial cleft and enables timely referral to a center with expertise.^4^

A wide range of sensitivities has been reported for the detection of oral clefts using prenatal transabdominal ultrasound at the mid‐trimester anomaly scan In low‐risk populations, prenatal detection rates for cleft lip with or without a cleft palate range from 9 to 100%.^5^ Detection rates for cleft palate without a cleft lip are poor with sensitivities ranging from 0 to 22%.^5^ In high‐risk populations, when 3D ultrasound techniques are used, detection rates have been reported to be as high as 100% for cleft lip and 86–90% for a cleft lip with a cleft palate.^5^

Screening for a cleft lip using axial and coronal planes is included in the International Society of Ultrasound in Obstetrics (ISUOG) practice guidelines for the mid‐trimester anomaly ultrasound.^6^ Screening for a cleft palate without a cleft lip is not part of these guidelines. The American Institute of Ultrasound in Medicine (AIUM) lists the upper lip as part of the components of a standard second trimester examination.^7^ They do note that imaging the palate may be included as part of a more detailed obstetric examination if indicated and refer to 3D techniques for visualizing the palate.^7^

Visualizing the palate directly on prenatal ultrasound is difficult as the palate is a dome‐shaped structure surrounded by osseous structures.^7^ Not all ultrasound machines have 3‐dimensional capacity (3D), and not all have the skill and time to assess the palate using 3D ultrasound techniques.

Embryological fusion of the secondary palate starts ventrally and proceeds dorsally. Therefore, if the uvula can be visualized, it implies an intact palate.^8^ In 2010 the “equals sign” which depicts the lateral borders of the uvula on 2‐dimensional ultrasound, in either the axial, coronal or sagittal plane has been described.^9^ It was reported that the uvula could be visualized in 90.7% of cases.^8^ If this sign could be validated and shown to be time efficient, it may be an option to consider adding it to the routine mid‐trimester scan.

The aim of this retrospective study was to quantify the time taken to obtain an image of the “equal sign” at the routine midtrimester fetal anomaly scan. The secondary objectives were to assess if there were any factors affecting the time and ease to identify the uvula. We also reviewed all cases where the equal sign was thought to be abnormal.

## Materials and Methods

### Population

A retrospective audit of a single ultrasound practice was performed. We included pregnant women who presented for assessment between October 1, 2013 and September 30, 2019 at Panorama Perinatology, Panorama Hospital, Cape Town, South Africa.

**Figure 1:**
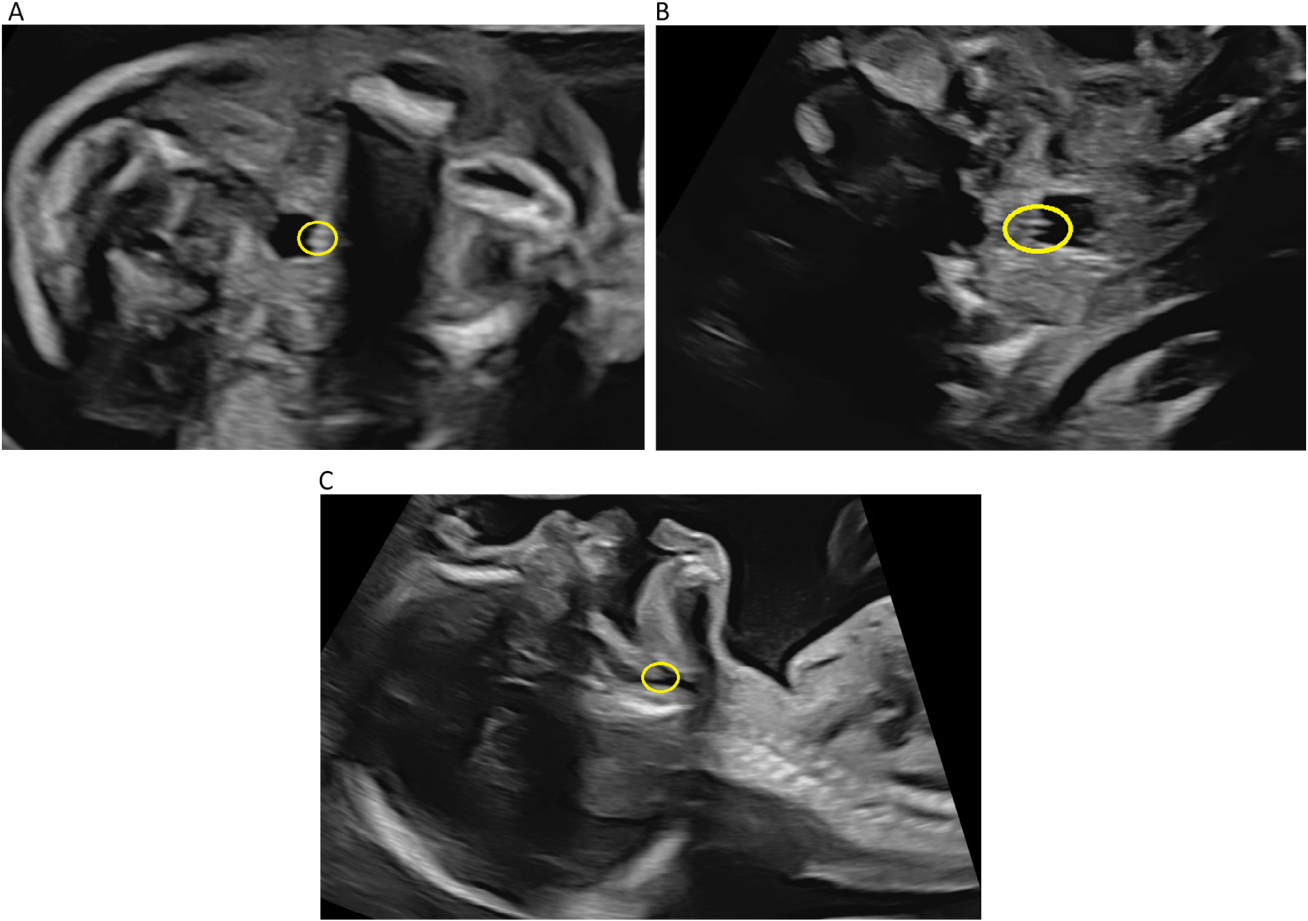
**A**, Axial image of the fetal head, caudal to the hard palate which demonstrates the uvula. The circle depicts the uvula (equal sign) in the pharynx. **B**, Coronal image of the fetal head demonstrating the uvula. The circle depicts the uvula (equal sign) in the pharynx. **C**, Midsagittal image of the fetal head demonstrating the uvula. The circle depicts the uvula (equal sign) in the pharynx.

**Figure 2:**
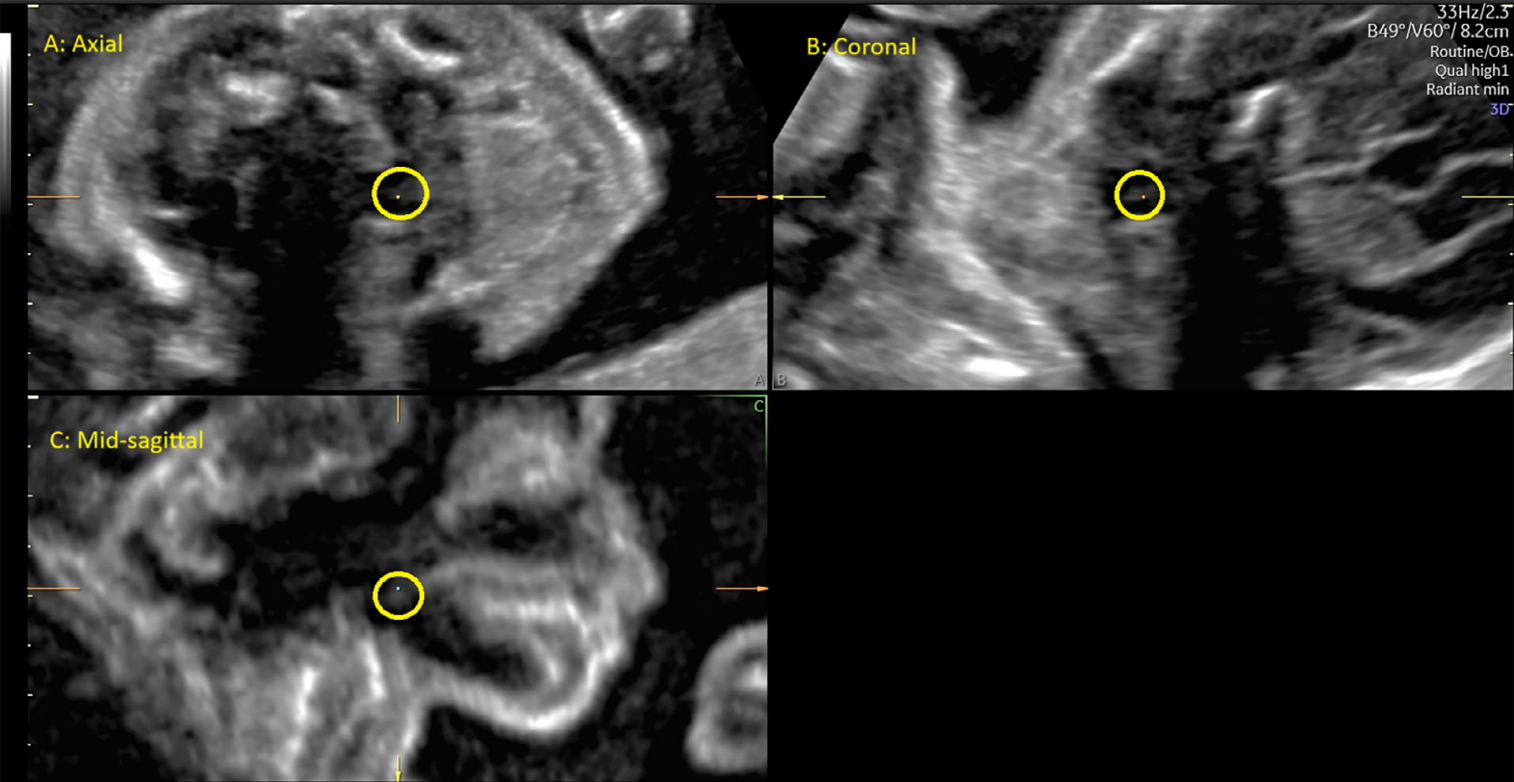
3D rendering showing the uvula (equal sign) in the axial (**A**), coronal (**B**), and sagittal (**C**) planes. The circle shows the equal sign.

The audit was restricted to cases referred for routine mid‐trimester screening between 18 weeks 0 days and 23 weeks 6 days. We excluded ultrasounds that were done for a second opinion or where other consultants were involved (eg, pediatric cardiologist or surgeon). We included singleton pregnancies irrespective of whether they resulted in a live birth, stillbirth, miscarriage or termination of pregnancy. All operators performing the mid‐trimester ultrasound had training to capture the image of the equal sign as described by Wilhelm and Borgers. Training was simple. A maternal fetal medicine specialist demonstrated where the uvula could be visualized in 3 planes in a live scan. The sonologists' images were then checked to ensure that the images were obtained correctly. A 2‐dimensional (2D) image was saved as either an axial, coronal or midsagittal image. The axial image was obtained by starting with a transverse section through the head at the level of the thalamus. The probe was moved parallel to this plane in a caudal direction until the nasopharynx and oropharynx were visualized. The uvula was then visualized as an equal sign (Figure 1A). The coronal image was obtained by acquiring a coronal view of the lips and nose. The probe was moved posteriorly, while maintaining the coronal position, until the uvula was visualized as an equal sign in the pharynx, superior to the epiglottis (Figure 1B). The midsagittal image was obtained by acquiring a profile plane. The probe was tilted in this plane until the uvula was visualized as an equal sign by following the soft palate posteriorly (Figure 1C). Imaging was performed transabdominally with either a GE Voluson E10 or E6 ultrasound machine in 2D. There were 2 maternal‐fetal medicine specialists and 2 radiographers who performed all the ultrasounds. A 3D image is provided as a reference to visualize the different planes (Figure 2). In our practice it is standard to image the uvula with 2D ultrasound.

### Sample Size

A sample size of 101 using an expected detection rate of 90% provided a power of 90% with an alpha error of 0.05 for an expected detection rate of 90% (Creative Research Systems, Sebastopol, CA, USA, 2019). A previous study reported the uvula could be detected in 90.7% of routine midtrimester ultrasound examinations.^9^ A random sample from all routine midtrimester ultrasounds was generated using Excel 2019, Microsoft, Redmond, WA, USA.

### Review of Images and Cases

The images for the 101 random cases were extracted from an Astraia electronic database (Astraia 1.25.2, Munich, Germany) by using the patient identification code on the excel sheet. Stored images were evaluated by 2 investigators (CC and AS) and consensus was reached as to whether the equals sign had been depicted in the axial, coronal or sagittal plane (Figure 1). Interobserver variability was assessed by comparing data extraction sheets from the 2 investigators for the presence or absence of the uvula on imaging. One investigator (AS) evaluated the images twice to determine intraobserver variability. Investigators were blinded to clinical outcomes.

We also selected all cases that had a cleft palate to review the cases with an abnormal equal sign.

### Outcomes

Baseline maternal characteristics including gravidity, parity, body mass index, and gestational age were collected. The time of day of the examination, whether the examination was performed by a maternal‐fetal medicine specialist or a sonographer, and length of the ultrasound examination were recorded. The duration of the scan was determined by calculating the time between the first recorded image and the last recorded image. The time to image the equal sign was calculated using the time interval between the penultimate image and the image depicting the equal sign. An image of the equals sign was considered to be in sequence if it was recorded while examining the face or brain. An image that was collected out of sequence implied that it was not possible to achieve the image of the uvula while examining the face or brain and that the examiner had to return later in the examination to visualize the uvula clearly. The image of the uvula was assessed for quality and was rated as either a good image or a dubious image. The image was classified as dubious if the image was angled (not a true axial, coronal, or midsagittal image) or if the equal sign depicting the uvula was not very clearly visible.

### Ethical Considerations

Ethics approval was obtained from the Health Research and Ethics Committee 2 at Stellenbosch University (N19/10/155, REC‐230208‐010). A waiver of consent was granted as this was a retrospective audit of ultrasound images and clinical information from an electronic database. No participants were contacted for the purpose of the study.

### Statistical Methods

Descriptive data are presented as means with standard deviations or medians with interquartile ranges for numerical variables and a number with percentages for categorical variables. Differences between sonographers and sonologists were compared using the Student t test for normally distributed continuous variables and the chi‐square Fisher's exact test for categorical variables. The interobserver and intraobserver variability are presented as percentage agreement and Cohen's kappa (*k*) was calculated with a value of *k* > 0.60 being considered substantial agreement. The statistical analysis was performed using IBM SPSS version 28.0.0.0.

## Results

Five thousand three hundred and one routine singleton fetal anomaly ultrasound examinations were performed during the time period and 101 cases were assessed. Baseline demographic characteristics are presented in Table 1.

**Table 1:** Baseline Characteristics on the 101 Cases that Were Assessed at the Midtrimester Fetal Anomaly Ultrasound for the Presence or Absence of the Uvula on Ultrasound

	Overall, *n* = 101	MFM, *n* = 59	Sonographer, *n*= 42
Mean maternal age years (SD)	32.8 (4.3)	33.5 (4.2)	31.9 (4.0)
Median gravidity [range]	2 [1–6]	2 [1–6]	2 [1–5]
Median parity [range]	1 [0–3]	1 [0–3]	1 [0–3]
BMI (SD)	26.1 (6.4)	26.8 (5.1)	25.2 (5.1)
BMI ≥30 (%)	21 (20.8)	16 (27.1)	5 (11.9)
Mean gestation in weeks at scan (SD)	20.8 (0.8)	20.9 (0.8)	20.7 (0.7)
Total scan time in minutes and seconds (SD)	33m36s (13m16s)	24m15s (6m31s)*	46m44s (8m6s)*

*n*, number; BMI, body mass index; MFM, maternal fetal medicine specialist; SD, standard deviation.

* *p* < .001 (2‐sided *p* value).

The uvula was visualized in 97% of cases (98/101). It was seen in sequence 88% (89/101) of the time and in the axial view in 83% of cases (81/98). Maternal fetal medicine specialists used multiple planes to visualize the uvula, while sonographers mostly used the axial plane. Maternal fetal medicine specialists imaged the uvula faster than sonographers, but the percentage of overall scan time to visualize the uvula was similar (1.5% versus 1.7%) (Table 2).

**Table 2:** Data on Visualization of the Uvula

	Overall, *n* = 101	MFM, *n* = 59	Sonographer, *n* = 42
Equal sign visualized, *n* (%)	98 (97.0)	56 (94.9)	42 (100)
Axial, *n* (%)	81 (82.7)	41 (69.5)*	40 (95.2)*
Coronal, *n* (%)	4 (4.0)	3 (5.1)	1 (2.4)
Sagittal, *n* (%)	9 (9.2)	9 (15.3)*	0 (0.0)*
Dubious, *n* (%)	4 (4.0)	3 (5.1)	1 (2.4)
In sequence, % (*n*)	88% (89/101)	88% (52/59)	88% (37/42)
Mean time to visualize equal sign in seconds (SD)	28 (24)	23 (22)*	36 (25)*
Percentage of total scan time in seconds to visualize equal sign (SD)	1.5 (1.4)	1.7 (1.6)	1.3 (0.8)

*n*, number; MFM, maternal fetal medicine specialist; SD, standard deviation.

* *p* < .01 (2 sided *p*‐value).

Interobserver variability was assessed for presence or absence of the uvula. In 6 cases (5.9%) there were differences. In 5 cases, 1 investigator saw the uvula clearly and the other did not. In 1 case 1 investigator thought the image was dubious and the other said the uvula was well seen. The percentage agreement for the interobserver variability where we compared whether the equal sign was seen or not was 95% with a Cohen's Kappa of 0.85. One investigator (AS) reviewed the images twice to determine the intraobserver variability. Three participants images had corrupted on the server and were not available for reassessment. There were 7 cases (7.1%) with different assessments. In 1 case the uvula was not seen in the first assessment but seen in the second assessment. In 2 cases the uvula was initially assessed to be dubious but in the second assessment it was well seen. In 1 case the uvula was reported as not seen and in the second assessment it was dubious. In 3 cases, the uvula was seen in the first assessment but not in the second assessment. The percentage agreement for the intraobserver variability where we compared whether the equal sign was seen or not seen was 97% with a Cohen's Kappa of 0.74. There were no statistically significant differences between ultrasounds performed by sonographers and maternal‐fetal medicine specialists except that maternal fetal medicine specialists had a significantly shorter median total scan time.

The body mass index (BMI) did not have a significant effect on the ability to obtain a clear image of the uvula. The uvula was clearly visualized in 93.8% (75/80) with a BMI less than 30 and 90.5% (19/21). In those with a BMI equal to or greater than 30 it was seen in 90.5% (19/21). The day of ultrasound scan did not have a significant effect on the ability to obtain a clear image of the uvula. The uvula was clearly visualized in 96.1% (49/51) of cases performed on a Monday to Wednesday and it was visualized in 90.0% (45/50) of cases performed on a Thursday to Saturday. If the ultrasound was performed after 1 pm there was a higher chance of not visualizing the uvula clearly (48/49 [98%] versus 46/52 [88.5%] % difference 9.5%; *p* < .01).

**Figure 3:**
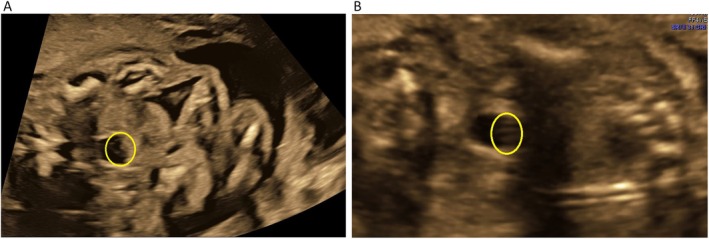
Images of abnormal uvulas. **A**, An abnormal equal sign in a fetus with an intact lip and maxilla. **B**, An abnormal equal sign associated with a cleft lip and palate.

In the 5301 routine midtrimester ultrasound examinations, there were 15 cases where the uvula was documented to be abnormal (Table 3). Ten cases had an intact lip (Figure 3A) and 5 had an associated cleft lip (Figure 3B). Cases with an intact lip with a cleft palate were often associated with other anomalies (Table 3). All cases where there was thought to be an abnormal equal sign were confirmed on third trimester scan or postnatally. There were no false positive diagnoses in these 15 cases. We did not have outcome data on the presence of cleft palate at birth in all 5301 cases and were therefore unable to determine how many false negative cases there were.

**Table 3:** Clinical Details on Cases with an Abnormal Equal Sign

Cases	Gestation at Diagnosis	Details on the Cleft	Other Ultrasound Findings	Genetic Testing and Family History	Diagnosis Confirmed	Outcome
Cleft lip and palpate						
1	19w0d	Bilateral cleft clip and palate	None	None	Confirmed at birth	Live birth
2	19w6d	Unilateral left cleft lip and palate. Maxilla intact	None	None	Confirmed at birth	live birth at 39w0d, 3310gm
3	20w5d	Unilateral left cleft lip, maxilla and palate	Hemivertebrae	None	Confirmed on ultrasound at 32 weeks	
4	22w5d	Unilateral right cleft lip and palate. Maxilla intact	None	None	Confirmed at birth	Live birth at 33w0d, 1970gm
5	20w4d	Unilateral left cleft lip, maxilla and palate	None	None	Confirmed at birth	Live birth
Intact lip with cleft palate						
6	20w0d	Midline cleft palate	None	None	Confirmed at birth	Live birth at 39w1d, 3300gm
7	21w4d	Large midline cleft palate	ARSA	Normal karyotype	Termination	
8	23w1d	Midline cleft palate	Mild ventriculomegaly	None	Confirmed at birth	Live birth
9	20w0d	Midline cleft palate	Lethal skeletal dysplasia	Normal FISH	Termination	
10	20w4d	Midline cleft palate	Micrognathia	None	Confirmed at birth	Live birth
11	20w2d	Cleft palate/ bifid uvula	Micrognathia	Mother born with a cleft palate	Confirmed on 32‐week ultrasound	
12	19w2d	Midline cleft palate	Duplex kidney with hydronephrosis and ectopic ureter	None	Confirmed at birth	Live birth at 38w5d, 3070gm
13	20w6d	Midline cleft palate	Multiple anomalies including ACC, hypoplastic cerebellum, VSD, rocker‐bottom feet	Deletion of a portion of chromosome 6	Termination	
14	20w6d	Midline cleft palate	Multiple anomalies	Trisomy 18	Termination	
15	19w2d	Midline cleft palate	Postaxial polydactyly	None	Confirmed on 31‐week ultrasound	

d, days; gm, grams; w, weeks; ACC, absent corpus callosum; ARSA, aberrant right subclavian artery; FISH, fluorescence in‐situ hybridization; VSD, ventricular septal defect.

## Discussion

### Principle Finding

Visualizing the uvula at the second trimester ultrasound is possible and feasible by both maternal fetal medicine specialists and sonographers in 2D. The uvula was clearly demonstrated in 97% of cases. The average time to visualize the uvula was 28.5 seconds and it increased the total ultrasound time by 1.5%. The uvula was seen in sequence the majority of times and was most often recorded in the axial plain. The mother's BMI or the day of the ultrasound did not influence whether the image was recorded but only 20% of our study had a BMI above 30 and the mean BMI of the group was 26. When ultrasounds performed in the morning were compared to those performed after 1 pm, the uvula was more often seen in the morning.

This is the first study assessing the feasibility of imaging of the uvula at the midtrimester fetal anomaly scan since it was first described in 2010.^9^ Assessing the equal sign allowed the detection of 15 cases with a possible abnormal soft palate. Of these, 5 had a cleft lip and should have been picked up on the midtrimester anomaly ultrasound. There were 10 cases of cleft palate without a cleft lip detected. All were confirmed on follow up ultrasound or postnatally so there were no false positive diagnoses, which is in keeping with previous research.^5^ The majority of these cases had other anomalies ranging from an aberrant right subclavian artery, post axial polydactyly, micrognathia, and mild ventriculomegaly to multiple anomalies and a lethal skeletal dysplasia. With the increased availability of microarray and whole exome sequencing, detecting these cases may have clinical implications in the future.

Our practice consists of 2 experienced sonographers and 2 maternal‐fetal medicine specialists. All sonographer images are reviewed by the maternal‐fetal medicine specialists and the time booked for a sonographer scan is 15 minutes longer resulting in a longer scan time. This may also explain why the detection rate for the sonographers was 100%. It may be more difficult for inexperienced practitioners to visualize the equal sign and may result in false positive cases. This was not the case in our practice. The uvula was more likely to be seen in the morning than the afternoon. This may be due to the operator becoming fatigued in the afternoon.

Limitations of the study include that we were unable to determine if any cases of cleft palate were missed, as we do not have a full outcome dataset on the presence of a cleft palate at birth. A cleft palate may also be missed in the postnatal period. The methods used to determine the amount of time needed to image the uvula are based on assumptions, and they may over or underestimate the time to get the image of the uvula.

In summary, it is feasible to image the equal sign at the midtrimester ultrasound with a low false positive detection rate. Maternal fetal medicine specialists and experienced sonologists could consider visualizing the uvula using the equal sign to assess for cleft palate. A large study with multiple sonographers, ideally in a low‐risk population, is needed to assess the utility of the equal sign for detecting cleft palate for routine scanning.

## Data Availability

The data that support the findings of this study are available from the corresponding author upon reasonable request.
